# The association of remnant cholesterol (RC) and interaction between RC and diabetes on the subsequent risk of hypertension

**DOI:** 10.3389/fendo.2022.951635

**Published:** 2022-08-25

**Authors:** Jie Wang, Qi Sun, Yu An, Jia Liu, Song Leng, Guang Wang

**Affiliations:** ^1^ Department of Endocrinology, Beijing Chao-Yang Hospital, Capital Medical University, Beijing, China; ^2^ Health Management Center, The Second Hospital of Dalian Medical University, Dalian, China

**Keywords:** remnant cholesterol, hypertension, dyslipidemia, cardiovascular disease, diabetes

## Abstract

**Purpose:**

Whether elevated remnant cholesterol (RC) is associated with hypertension (HTN) and whether elevated RC interacts with diabetes on the subsequent risk of HTN have not been illustrated. Thus, this study is aimed to investigate the associations and interactions of RC, diabetes, and the management of cardiovascular risk factors with the risk of incident HTN in a Chinese population.

**Patients and methods:**

This cohort study included 42,994 individuals who participated in the routine health check-up from April 2016 to August 2020 and follow-ups from April 2017 to August 2021 at the Medical Examination Center of Beijing Chao-Yang Hospital. RC was divided into quintiles as follows: the < 20% group, the 20–39% group, the 40–59% group, the 60–79% group, and the ≥ 80% group. This study finally included 17,006 participants who were free from HTN at baseline.

**Results:**

This study had 1,861 (10.90%) HTN occurred, 205 (5.30%) in the first quintile of RC, 335 (8.98%) in the second quintile of RC, 388 (11.17%) in the third quintile of RC, 420 (13.42%) in the fourth quintile of RC, and 513 (17.91%) in the fifth quintile of RC. Compared with participants in the first quintile of RC, participants in the fifth quintile of RC showed a greater risk of HTN events among participants with diabetes [hazard ratio (HR), 4.95; 95% confidence interval (CI), 1.05–23.39; P = 0.0432) than among participants without diabetes (HR, 1.67; 95% CI, 1.26–2.22, P = 0.0004; P for interaction = 0.0420). Compared with participants without diabetes, participants with diabetes who have the ideal management of RC and other risk factors showed no excess risk of HTN.

**Conclusions:**

Elevated RC is significantly predictive of HTN among the diabetic population. RC and diabetes interacted with each other on the subsequent risk of HTN, and the desired management of RC, glucose, and cardiovascular risk factors on HTN risk was quite favorable.

## Introduction

Hypertension (HTN), type 2 diabetes mellitus (T2DM), and dyslipidemia are established risk factors for cardiovascular diseases (CVDs) internationally (1, 2). HTN in the non-diabetic and diabetic population has been confirmed associated with the increased risk of adverse cardiovascular events. It is reported that the coexistence of dyslipidemia, T2DM, and HTN is often observed, and over 50% of hypertensive patients have been diagnosed with dyslipidemia in clinical practice (3, 4). Moreover, CVDs remain the most common cause of death in people with T2DM and HTN. Therefore, it is of great significance to prevent or reduce the incidence of HTN, together with risk factor management such as the management of blood glucose and cholesterol, contributing to the reduction of CVDs in the diabetic population. The sensitive lipid parameters might possess great clinical value, due to the ability to stratify risk for the diabetic population.

Dyslipidemia has been regarded as an accelerator for the increased risk of T2DM, HTN, and CVDs. Evidence from epidemiological and genetic research has engendered new interests that demonstrate that abnormal triglyceride (TG), low-density lipoprotein cholesterol (LDL-C), lipoprotein(a) [Lp(a)], or TG-rich lipoproteins (TGRLs) are additional risk of atherosclerosis CVD (ASCVD)–related death (5–8). A growing body of studies pointed out that LDL-C is associated with CVDs risk and that lowering LDL-C to desired range could reduce the risk of CVDs due to the achieved reduction in LDL-C (9), and other studies suggested that lowering TG reduces the risk of CVDs, which are equal to LDL-C–lowering therapies (10). A follow-up study of 5,971 women found a significant association between lipid parameters and HTN, and studies based on adolescents reached similar results (11, 12). However, recurrent CVD is still a major cause of mortality worldwide although achieving an optimal LDL-C level in clinical practice (13, 14).

Recently, TGRLs and their cholesterol content, known as remnant cholesterol (RC), have been revealed to contribute to the residual risk of CVDs (15, 16). A causal relationship between RC and ischaemic heart disease has been reported, and in addition, high RC levels have been linked to mortality and adverse cardiovascular events (17, 18). Emerging data have reported a genetic association between increased RC and CVDs (19, 20). Although the direct measurement of RC has been developed, it is still difficult to be operational for clinical practice. Thus, the calculation RC has been proposed as a clinically feasible tool (21). Although previous clinical studies have observed a significant association between RC and cardiovascular events, studies about the predictive implications of RC for HTN, especially in the context of T2DM, are inadequate (17). Thus far, it remains unknown whether RC is superior to other individual lipid parameters in predicting the incidence of HTN and whether the RC-related HTN risk differs between diabetic and non-diabetic populations.

To fill these gaps in knowledge, we aimed to assess the bidirectional interactions of high RC levels and individual lipid parameters with diabetes status on the subsequent risk of HTN and investigate the association of cardiovascular risk factor management in diabetes with the subsequent risk of HTN.

## Methods and materials

### Study population

**Figure 1:**
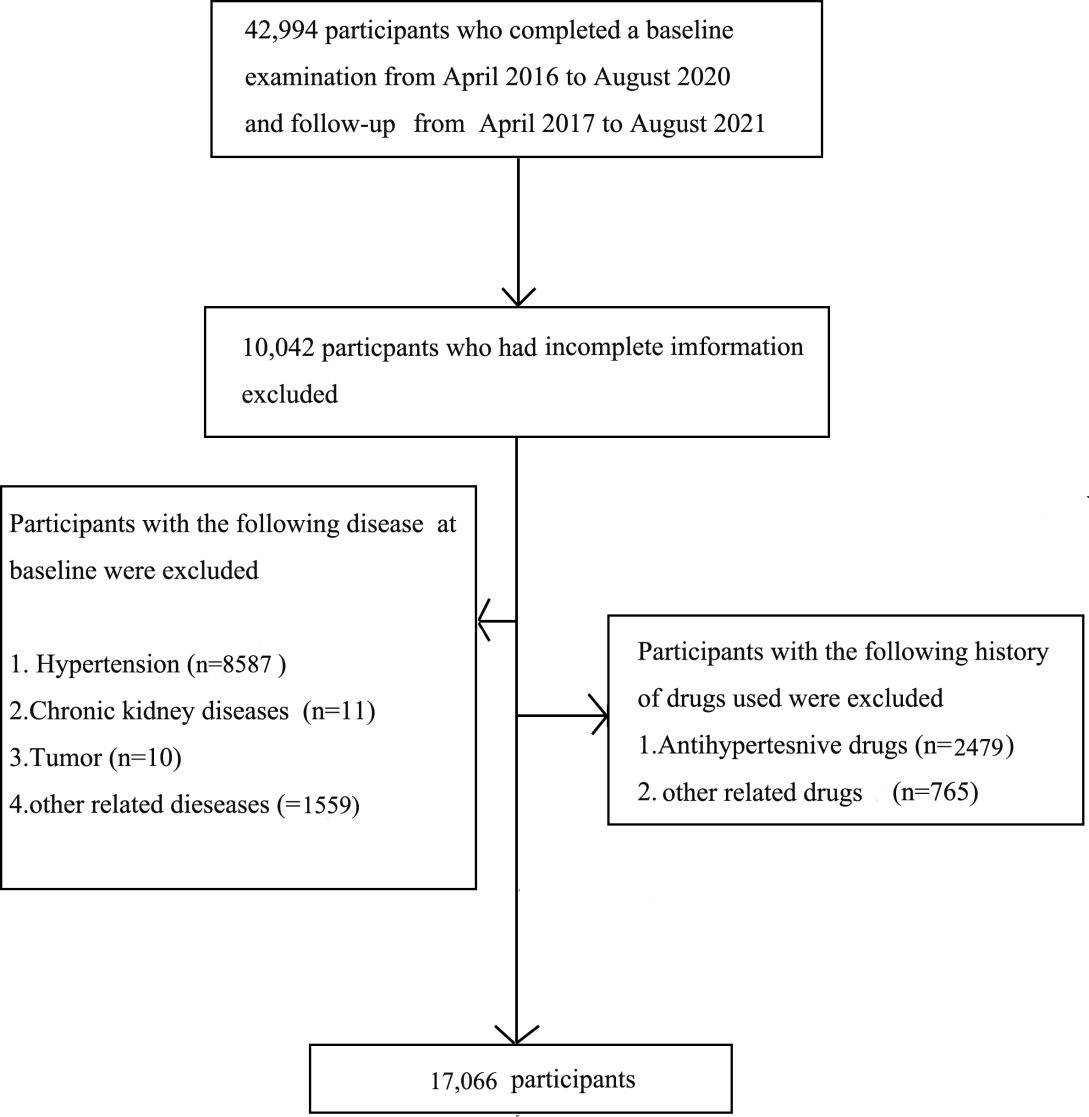
Flow chart of the selection study participants.

The present study included 42,994 individuals who participated in the routine health checkup from April 2016 to August 2020 and follow-ups from April 2017 to August 2021 at the Medical Examination Center of Beijing Chao-Yang Hospital. The exclusion criteria are as follows: the previous history of HTN, currently diagnosis with HTN, other previous history of related chronic diseases, using related drugs, missing detailed data, and/or included outliers. Finally, 17,066 individuals were recruited (**Figure 1**). This study was approved by the Ethics Committee of the Beijing Chao-Yang Hospital affiliated with Capital Medical University. Written informed consent was obtained before the study.

### Baseline data collection

All participants who were recruited underwent detailed clinical examination by experienced physicians and anthropometric measurements. The medication history was collected on the basis of the self-reported history of T2DM, HTN, dyslipidemia, kidney diseases, hepatic diseases, the current use of drugs, and drinking and smoking habits. Blood pressure was recorded twice by the same staff when participants were in a seated position for a 5-min rest using a standard sphygmomanometer.

Blood samples were obtained from the antecubital vein after ≥8 h of overnight fasting and stored at −80°C until analysis. Fasting plasma glucose (FPG), TG, TC, HDL-C, LDL-C, aspartate aminotransferase (AST), alanine aminotransferase (ALT), and creatinine (Cr) were measured by colorimetric enzymatic assays using a biochemical auto-analyzer (Hitachi 7170), and uric acid (UA) was measured using a Siemens Advia 2400 biochemical analyzer (Siemens Healthcare Diagnostics Inc., Tarrytown, New York, USA) as previously described (22).

The estimated glomerular filtration rate (eGFR) was expressed in milliliters per minute per 1.73 m^2^ using the following formula: eGFR = 175 × (serum Cr in mg/dL)^−1.154^ × age^−0.203^ × (0.742 for women) × (1.212 if African American) (23). RC was evaluated using the formula: RC = TC − HDL-C − LDL-C (24).

### Follow-up examination

The endpoint outcome of this current study was the incidence of HTN during the follow-up period. Participants were followed up for a median of 1.1 years using annual medical checkup data that are collected between April 2017 and August 2021.

### Definition of variables

RC was divided into five groups: the < 20% group, the 20%–39% group, the 40%–59% group, the 60%–79% group, and the ≥ 80% group, based on the quintile division of the participants. According to the Chinese guideline for the management of dyslipidemia in adults (revised in 2016), lipid parameters were categorized into several groups as follows: TG: normal: < 1.7 mmol/L, borderline: 1.7–2.3 mmol/L, high: ≥ 2.3 mmol/L; TC: normal: < 5.2 mmol/L, borderline: 5.2–6.2 mmol/L, high: ≥ 6.2 mmol/L; HDL-C: desired: ≥ 1.0 mmol/L, low: < 1.0 mmol/L; 4. LDL-C: ideal < 2.6 mmol/L, borderline: 3.4–4.1 mmol/, high: ≥ 4.1 mmol/L (25). HTN was defined as systolic blood pressure (SBP) ≥ 140 mmHg or diastolic blood pressure (DBP) ≥ 90 mmHg or currently taking antihypertensive medication or diagnosed with HTN by clinicians during the follow-up period. T2DM was defined as a self-reported physician-diagnosis history, currently treated with insulin or oral hypoglycemic agents, or an FBG ≥ 7.0 mmol/L (26). Fatty liver disease was defined as hepatic steatosis confirmed by hepatic ultrasonography. Body mass index (BMI) was calculated using the following formula: BMI = body weight/height^2^ (kg/m^2^). Participants were divided into two groups according to their smoking frequency: no, never or have already quit smoking; yes, regularly smoking cigarettes in the past years. Participants were divided into two groups according to their alcohol intake frequency: no, never or have already quit drinking; yes, regularly drinking more than once a week in the past years.

### Statistical analysis

Empower(R) (www.empowerstats.com, X&Y Solutions Inc., Boston, MA) and R (http://www.Rproject.org) were used to perform the statistical analyses. The hazard ratios (HR) and corresponding 95% confidence intervals (95% CI) were calculated. All statistical tests were two-sided, and P-values < 0.05 were considered statistically significant.

The normality of the variables was assessed using the Kolmogorov–Smirnov test. Continuous variables with a non-normal distribution were compared using Kruskal–Wallis test and were shown as median (Q1–Q3). Continuous variables with a normal distribution were compared using Student’s t-tests and were shown as means ± the standard deviations (SD). Categorical variables were presented as n% and were compared using the χ2 test. Cox proportional hazards analysis was performed to evaluate the associations of RC and individual lipid parameters with HTN among participants with and without diabetes, in multivariate settings with adjustments for potential confounding factors. Three models were built to investigate and compare the associations between RC, lipid parameters, and HTN in the context of T2DM. Model 0 was unadjusted. Model 1 was adjusted for age and sex. Model 2 was adjusted for age, sex, BMI, TG, TC, LDL-C, HDL-C, UA, ALT, AST, eGFR, FBG, smoking habits, and drinking habits. Associations of diabetes with HTN events across RC quintiles were also established. Multiplicative interactions between RC quintiles and diabetes on HTN events were also tested in the models. Associations of individual conventional risk factor management with HTN events among participants with diabetes according to BMI groups (27)(underweight: < 18.5 kg/m^2^; normal weight: 18.5–24 kg/m^2^; overweight: 24–28 kg/m^2^; obesity: BMI of ≥ 28 kg/m^2^), LDL-C (ideal, <2.6 mmol/L; high, ≥2.6 mmol/L), HDL-C (low, <1.0 mmol/L; desired, ≥1.0 mmol/L), UA (ideal, <420 μmol/L; abnormal, ≥420μmol/L), RC quintiles, and fatty liver disease (no and yes), as compared with participants without diabetes were analyzed.

## Results

### Baseline characteristics of the study population

The baseline characteristics of 17,066 participants (9,465 men and 7,601 women) with a mean age (Q1–Q3) of 37.0 (31.0–47.0) years were shown in **Table 1**. With the increase in RC levels, BMI, SBP, DBP, FBG, TC, TG, LDL-C, ALT, AST, and UA significantly increased, and HDL-C and eGFR significantly decreased. The percentiles of smoking and drinking habits were highest in the highest quintile of RC (Q5). In addition, the incidence of HTN 513 (17.91%) during the follow-up period was highest in participants with the highest quintile of RC (Q5).

**Table 1:** Characteristics of the study population by RC quintiles.

	Total	RC quantiles					
		Q1	Q2	Q3	Q4	Q5	P-value
N	17,066	3,612	3,486	3,246	3,486	3,236	
Age, years	37.0 (31.0–47.0)	33.0 (29.0–41.0)	36.0 (30.0–46.0)	39.0(32.0–49.0)	43.0 (34.0–50.0)	44.0 (35.0–52.0)	<0.001
BMI, kg/m^2^	23.5 (21.2–25.9)	22.0 (21.0–24.2)	22.9 (20.8–25.2)	23.6(21.5–26.0)	24.4 (22.3–26.6)	25.3 (23.2–27.5)	<0.001
SBP, mm/Hg	121 (112 –129)	118 (110–126)	120 (111–128)	121 (112–129)	122 (113–130)	124 (116–131)	<0.001
DBP, mm/Hg	73 (67–79)	7 1(65–77)	72 (66–78)	73 (67–79)	74 (68–80)	76 (70–82)	<0.001
FBG,							
mmol/L	5.33 (5.06–5.65)	5.19 (4.94–5.46)	5.26 (5.01–5.54)	5.34 (5.06–5.65)	5.41 (5.15–5.75)	5.53 (5.23–5.93)	<0.001
TC,							
mmol/L	4.73 (4.20–5.33)	4.05 (3.68–4.46)	4.49 (4.12–4.89)	4.82 (4.38–5.24)	5.16 (4.72–5.66)	5.67 (5.10–6.25)	<0.001
TG,							
mmol/L	1.41 (1.01–1.96)	1.01 (0.78–1.31)	1.23 (0.92–1.56)	1.44 (1.08–1.83)	1.78 (1.34–2.21)	2.56 (1.83–3.45)	<0.001
HDL-C,							
mmol/L	1.29 (1.09–1.52)	1.42 (1.23–1.63)	1.37 (1.16–1.60)	1.27 (1.09–1.50)	1.21 (1.04–1.45)	1.09 (0.93–1.30)	<0.001
LDL-C,							
mmol/L	2.50 (2.08–2.95)	2.07 (1.75–2.42)	2.36 (2.02–2.72)	2.58 (2.22–2.97)	2.79 (2.42–3.18)	2.94 (2.44–3.41)	<0.001
ALT,							
U/L							
	19 (13–28)	15(11–21)	17 (12–24)	19 (14–27)	22 (16–32)	26 (18–39)	<0.001
AST,							
U/L							
	20 (17–24)	18 (16–21)	19 (16–23)	20 (17–24)	21 (18–25)	22 (19–27)	<0.001
eGFR, ml/min·1.73 m^2^	85.99 (71.67–106.62)	97.64 (78.15–114.23)	90.94 (73.86–110.82)	84.57 (71.07–105.31)	80.77 (70.13–99.02)	77.76 (67.99–92.64)	<0.001
UA, μmol/L	339 (278–407)	303(255–364)	321 (269–387)	341 (279–408)	359(298–420)	393 (327–461)	<0.001
RC,							
mmol/L	0.86 (0.67–1.11)	0.55 (0.47–0.60)	0.74 (0.70–0.78)	0.90 (0.85–0.94)	1.09 (1.04–1.16)	1.48 (1.34–1.72)	<0.001
SEX, %							<0.001
men	9, 465 (55.46%)	1, 546 (40.48%)	1, 792 (48.31%)	1, 969 (56.63%)	2, 027 (64.29%)	2, 131 (73.28%)	
women	7, 601 (44.54%)	2, 273 (59.52%)	1, 917 (51.69%)	1, 508 (43.37%)	1, 126 (35.71%)	777 (26.72%)	
Smoking, %							<0.001
No	16, 865 (98.82%)	3, 824 (98.90%)	3, 706 (99.33%)	3, 438 (98.94%)	3, 080 (98.43%)	2, 817 (98.29%)	
Yes	201 (1.18%)	41 (1.10%)	25 (0.67%)	37 (1.06%)	49 (1.57%)	49 (1.71%)	
Drinking, %							0.005
No	17, 011 (99.68%)	3, 857 (99.79%)	3, 723 (99.79%)	3, 466 (99.77%)	3, 119 (99.65%)	2, 846 (99.30%)	
Yes	55 (0.32%)	8 (0.21%)	8 (0.21%)	8 (0.23%)	11 (0.35%)	20 (0.70%)	
Baseline diabetes, %							<0.001
No	15, 669 (91.81%)	3, 601 (96.18%)	3, 467 (95.33%)	3, 194 (92.31%)	2, 861(90.71%)	2, 546 (92.90%)	
Yes	1,397 (8.19%)	143 (3.82%)	170 (4.67%)	266 (7.69%)	293 (9.29%)	525 (17.10%)	
HTN incidence, %							<0.001
No	15, 205 (89.10%)	3, 662 (94.70%)	3, 395 (91.02%)	3, 086 (88.83%)	2, 710 (86.58%)	2, 352 (82.09%)	
Yes	1,861 (10.90%)	205 (5.30%)	335 (8.98%)	388 (11.17%)	420 (13.42%)	513 (17.91%)	

Data were mean ± SD or median (IQR) for skewed variables or numbers (proportions) for categorical variables.

HTN, hypertension; BMI, body mass index; SBP, systolic blood pressure; DBP, diastolic blood pressure; FBG, fasting plasma glucose; ALT, alanine transferase; AST, aspartate transferase; TG, triglyceride; TC, high cholesterol; LDL-C, low-density lipoprotein cholesterol; HDL-C, high-density lipoprotein cholesterol; eGFR, estimated glomerular filtration rate; UA, uric acid; RC, remnant cholesterol.

### Associations of RC and lipid parameters with HTN events

**Figure 2:**
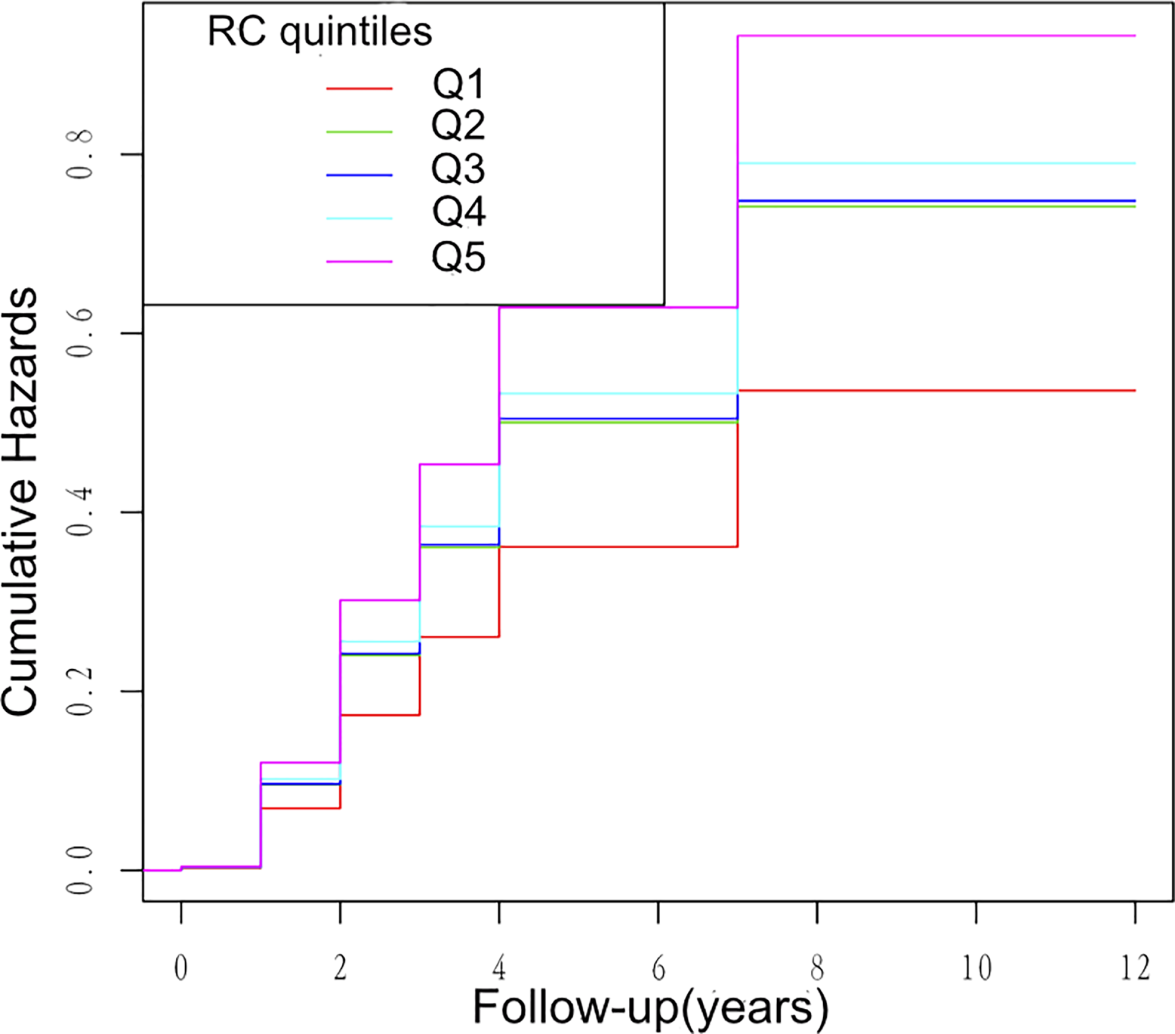
Kaplan–Meier analysis between RC quintiles and HTN events.

Multivariate cox regression models were constructed to access the predictive ability of RC and individual lipid profiles with the incidence of HTN. During 1.1 years of follow-up, 1,861 HTN events were documented. **Table 2** shows the HR and 95% CI of the incidence of HTN with the groups of RC quintiles, TG, TC, HDL-C, and LDL-C in the total population within three models. As shown in **Table 2**, RC and other lipid parameters all presented a significant association with HTN in the non-adjusted model and Model 1 adjusting for age and sex. Further adjustments for ALT, AST, FBG, UA, TC, TG, HDL-C, LDL-C, eGFR, FBG, smoking, and drinking habits in Model 2, only RC quintiles and TG groups remained significantly associated with HTN. Compared with participants with the first quintile of RC (Q1), participants with the highest quintile of RC (Q5) had a significantly higher hazard of the incidence of HTN. Of note, the fifth quintile of RC showed a stronger association with HTN than TG groups, suggesting the predictive ability of high RC for HTN risk (fifth quintile of RC: HR, 1.69; 95% CI, 1.31–2.18; p < 0.0001; TG groups: 1.7 ≤ TG < 2.3 mmol/L: HR, 1.21; 95% CI, 1.07–1.37; P = 0031; TG ≥ 2.3 mmol/L: HR, 1.17; 95% CI, 1.00–1.36; P = 0.0480). Kaplan–Meier analysis between RC quintiles and HTN events showed similar results (**Figure 2**).

**Table 2:** Association of RC, lipid parameters with HTN events.

Variable	Cases of HTN	Non-adjusted	Adjust 1	Adjust 2
		HR (95% CI) P-value	HR (95% CI) P-value	HR (95% CI) P-value
RC quantiles				
Q1	205	1.0	1.0	1.0
Q2	335	1.69 (1.41, 2.02) < 0.0001	1.43 (1.20, 1.72) < 0.0001	1.37 (1.14, 1.66) 0.0008
Q3	388	2.10 (1.77, 2.51) < 0.0001	1.53 (1.28, 1.82) < 0.0001	1.42 (1.17, 1.72) 0.0003
Q4	420	2.53 (2.13, 3.00) < 0.0001	1.67 (1.40, 1.99) < 0.0001	1.51 (1.22, 1.85) 0.0001
Q5	513	3.37 (2.85, 3.98) < 0.0001	2.00 (1.69, 2.37) < 0.0001	1.69 (1.31, 2.18) < 0.0001
TC,mmol/L				
<5.2	1142	1.0	1.0	1.0
≥5.2, <6.2	536	1.42 (1.28, 1.58) < 0.0001	1.17 (1.05, 1.30) 0.0033	1.05 (0.90, 1.22) 0.5745
≥6.2	183	1.72 (1.47, 2.02) < 0.0001	1.30 (1.10, 1.53) 0.0016	1.04 (0.79, 1.37) 0.7597
TG,mmol/L				
<1.7	885	1.0	1.0	1.0
≥1.7, <2.3	495	1.75 (1.56, 1.96) < 0.0001	1.40 (1.24, 1.57) < 0.0001	1.21 (1.07, 1.37) 0.0031
≥2.3	481	2.25 (2.01, 2.52) < 0.0001	1.61 (1.44, 1.81) < 0.0001	1.17 (1.00, 1.36) 0.0480
HDL-C,mmol/L				
≥1	1444	1.0	1.0	1.0
<1	417	0.61 (0.54, 0.68) < 0.0001	0.79 (0.70, 0.88) < 0.0001	0.97 (0.84, 1.13) 0.7269
LDL-C, mmol/L				
<3.4	1588	1.0	1.0	1.0
≥3.4, <4.1	231	1.55 (1.35, 1.79) < 0.0001	1.23 (1.06, 1.41) 0.0053	1.03 (0.86, 1.23) 0.7633
≥4.1	42	1.25 (0.92, 1.72) 0.1582	1.01 (0.74, 1.38) 0.9642	0.75 (0.52, 1.10) 0.1399

Model 0: Adjusted for no confounding factors.

Model 1: Adjusted for age and sex.

Model 2: Adjusted for age, sex, BMI, ALT, AST, eGFR, FBG, TC, TG, HDL-C, LDL-C, UA, smoking habits, and drinking habits.

### Association of RC and lipid parameters with HTN incidence among participants with and without diabetes

As shown in **Table 3**, compared with participants with the first quintile of RC (Q1), participants with the highest quintile of RC (Q5) had a significantly 4.95-fold risk of HTN events in the diabetic population (fifth quintile of RC: HR, 4.95; 95% CI, 1.05–23.29; P = 0.0432), and the HRs for comparison of high RC level (Q5) with low RC level (Q1) were 1.67 (95% CI, 1.26–2.22; P = 0.0004) in the non-diabetic population. It is noteworthy that a significant interaction between RC quintiles and diabetes on subsequent risk of HTN incidence was also investigated (p for interaction in model 2 = 0.0420). We also observed a significant association between TG and HTN (1.7 ≤ TG < 2.3 mmol/L: HR, 1.24; 95% CI, 1.09–1.40; P = 0.0011) among participants without diabetes, but no significant association was found in participants with diabetes (p for interaction in model 2 = 0.0862). Importantly, it is elevated RC but not TG that remained associated with HTN events among participants with diabetes and participants without diabetes, suggesting the superior predictive ability of RC for HTN risk.

**Table 3:** Association of RC, lipid parameters with HTN incidence among participants with and without diabetes.

Variable	Non-adjusted	P for interaction	Adjust 1	P for interaction	Adjust 2	P for interaction
	HR (95% CI) P-value		HR (95% CI) P-value		HR (95% CI) P-value	
Diabetes						
RC quantiles		0.4891		0.1158		0.0420
Q1	1.0		1.0		1.0	
Q2	1.68 (0.42, 6.72) 0.4631		1.52 (0.38, 6.10) 0.5542		1.85 (0.34, 9.95) 0.4737	
Q3	3.23 (0.95, 10.97) 0.0600		2.86 (0.84, 9.74) 0.0918		4.06 (0.91, 18.15) 0.0668	
Q4	4.23 (1.28, 13.98) 0.0180		3.94 (1.19, 13.04) 0.0245		4.82 (1.08, 21.63) 0.0398	
Q5	4.27 (1.33, 13.73) 0.0147		3.73 (1.16, 11.98) 0.0273		4.95 (1.05, 23.29) 0.0432	
TC, mmol/L		0.6699		0.6825		0.7196
<5.2	1.0		1.0		1.0	
≥5.2, <6.2	1.12 (0.71, 1.77) 0.6113		1.05 (0.67, 1.66) 0.8242		0.82 (0.47, 1.43) 0.4812	
≥6.2	1.41 (0.84, 2.36) 0.1951		1.40 (0.83, 2.36) 0.2110		0.84 (0.38, 1.84) 0.6614	
TG, mmol/L		0.2689		0.1974		0.0862
<1.7	1.0		1.0		1.0	
≥1.7, <2.3	1.10 (0.59, 2.03) 0.7654		1.11 (0.60, 2.04) 0.7471		1.04 (0.54, 2.00) 0.9088	
≥2.3	1.88 (1.21, 2.91) 0.0048		1.85 (1.20, 2.87) 0.0058		1.69 (0.96, 2.98) 0.0675	
HDL-C, mmol/L		0.3399		0.7498		0.8457
≥1	1.0		1.0		1.0	
<1	0.79 (0.53, 1.19) 0.2580		0.77 (0.51, 1.16) 0.2167		1.13 (0.68, 1.86) 0.6324	
LDL-C, mmol/L		0.6915		0.3571		0.2153
<3.4	1.0		1.0		1.0	
≥3.4, <4.1	1.60 (0.96, 2.68) 0.0703		1.59 (0.95, 2.65) 0.0767		1.67 (0.86, 3.25) 0.1331	
≥4.1	0.92 (0.34, 2.51) 0.8697		0.91 (0.33, 2.50) 0.8621		1.04 (0.29, 3.74) 0.9504	
No Diabetes						
RC quantiles						
Q1	1.0		1.0		1.0	
Q2	1.69 (1.41, 2.02) < 0.0001		1.44 (1.20, 1.72) < 0.0001		1.37 (1.13, 1.67) 0.0017	
Q3	2.06 (1.72, 2.46) < 0.0001		1.50 (1.25, 1.79) < 0.0001		1.40 (1.14, 1.72) 0.0014	
Q4	2.44 (2.05, 2.91) < 0.0001		1.62 (1.35, 1.93) < 0.0001		1.52 (1.21, 1.90) 0.0003	
Q5	3.24 (2.73, 3.84) < 0.0001		1.94 (1.63, 2.31) < 0.0001		1.67 (1.26, 2.22) 0.0004	
TC, mmol/L						
<5.2	1.0		1.0		1.0	
≥5.2, <6.2	1.42 (1.27, 1.58) < 0.0001		1.18 (1.05, 1.31) 0.0036		1.04 (0.89, 1.22) 0.5919	
≥6.2	1.67 (1.41, 1.98) < 0.0001		1.26 (1.06, 1.50) 0.0075		0.98 (0.75, 1.28) 0.8871	
TG, mmol/L						
<1.7	1.0		1.0		1.0	
≥1.7, <2.3	1.77 (1.58, 1.99) < 0.0001		1.41 (1.25, 1.58) < 0.0001		1.24 (1.09, 1.40) 0.0011	
≥2.3	2.17 (1.93, 2.45) < 0.0001		1.56 (1.39, 1.76) < 0.0001		1.16 (0.99, 1.36) 0.0627	
HDL-C, mmol/L						
≥1	1.0		1.0		1.0	
<1	0.62 (0.55, 0.70) < 0.0001		0.80 (0.71, 0.90) 0.0003		0.99 (0.86, 1.14) 0.8707	
LDL-C, mmol/L						
<3.4	1.0		1.0		1.0	
≥3.4, <4.1	1.52 (1.31, 1.76) < 0.0001		1.20 (1.03, 1.39) 0.0172		1.01 (0.84, 1.21) 0.9382	
≥4.1	1.24 (0.89, 1.73) 0.2030		1.00 (0.72, 1.40) 0.9885		0.71 (0.48, 1.06) 0.0919	

Model 0: Adjusted for no confounding factors.

Model 1: Adjusted for age and sex.

Model 2: Adjusted for age, sex, BMI, ALT, AST, eGFR, FBG, TC, TG, HDL-C, LDL-C, UA, smoking habits, and drinking habits.

### Association of diabetes with HTN incidence among participants with RC quintiles

Diabetes was also significantly associated with an increased risk of HTN, and this association was more prominent among participants with high RC levels (Q4 and Q5), as shown in **Table 4**. The multivariable-adjusted HRs for the incidence of HTN associated with diabetes were 1.80 (95% CI 1.20–2.70, P = 0.0048) among participants with the fourth quintile of RC (Q4), and 1.47 (95% CI 1.08–2.00, P = 0.0148) among those with the fifth quintile of RC (Q5) (p for interaction in model 2 = 0.0212), indicating the bidirectional interaction of diabetes with RC on the subsequent risk of HTN.

**Table 4:** Association of diabetes with HTN incidence among participants with RC quintiles.

Variable	RC quantiles, mmol/L					
	Q1	Q2	Q3	Q4	Q5	P for interaction (treat RC quintiles as continuous)
Non-adjusted						
HR (95% CI) P-value						0.4395
Without Diabetes	1.0	1.0	1.0	1.0	1.0	
Diabetes	1.34 (0.43, 4.20) 0.6131	1.33 (0.59, 2.99)0.4840	2.11 (1.31, 3.38) 0.0021	2.33 (1.56, 3.46) < 0.0001	1.77 (1.31, 2.39) 0.0002	
Adjust 1						
HR (95% CI) P-value						0.0581
Without Diabetes	1.0	1.0	1.0	1.0	1.0	
Diabetes	0.59 (0.19, 1.86) 0.3712	0.67 (0.30, 1.52) 0.3390	1.35 (0.84, 2.18) 0.2194	1.77 (1.19, 2.64) 0.0051	1.45 (1.07, 1.97) 0.0167	
Adjust 2						
HR (95% CI) P-value						0.0212
Without Diabetes	1.0	1.0	1.0	1.0	1.0	
Diabetes	0.59 (0.19, 1.87) 0.3717	0.64 (0.28, 1.46) 0.2913	1.37 (0.84, 2.22) 0.2035	1.80 (1.20, 2.70) 0.0048	1.47 (1.08, 2.00) 0.0148	

Model 0: Adjusted for no confounding factors.

Model 1: Adjusted for age and sex.

Model 2: Adjusted for age, sex, BMI, ALT, AST, eGFR, TC, TG, HDL-C, LDL-C, UA, smoking habits, and drinking habits.

### Association of individual risk factors and HTN incidence among participants with diabetes, as compared with participants without diabetes

Stratified analyses were performed to thoroughly confirm the association and predictive value of elevated RC for incident HTN, as shown in **Table 5**. Compared with participants without diabetes, participants with diabetes who had controlled risk factors well exhibited no additional risk for HTN events, including those with BMI < 24 kg/m^2^, LDL-C< 2.6 mmol/L, HDL-C ≥ 1.0 mmol/L, UA < 420 μmol/L, low RC levels (Q1–Q3), and without fatty liver diseases, suggesting the beneficial effects of controlling risk factors in the diabetic population. To be noted, compared with participants without diabetes, the hazards of HTN were more prominent among participants with diabetes who had poor management of the following risk factors (24 ≤ BMI < 28 kg/m^2^: HR, 1.37; 95% CI, 1.02–1.84; P = 0.0354; LDL-C ≥ 2.6 mmol/L: HR, 1.30; 95% CI, 0.99–1.71; P = 0.0625; UA 420 ≥ μmol/L: HR, 1.68; 95% CI, 1.19–2.38; P = 0.0030; RC from the fourth to the fifth quintiles: Q4: HR, 1.69; 95% CI, 1.12–2.56; P = 0.0132; Q5: HR, 1.48; 95% CI, 1.08–2.02; P = 0.0147; with fatty liver diseases: HR, 1.36; 95% CI, 1.04–1.77; P = 0.0231), indicating the importance of conventional risk factor management on HTN incidence in the diabetic population.

**Table 5:** Associations of risk factors and HTN among participants with diabetes compared with those without diabetes.

Variable	Cases	HTN
		HR (95% CI) P-value
No Diabetes	1664	1.0
Diabetes		
BMI, kg/m^2^		
<18.5	10	1.52 (0.53, 4.38) 0.4413
≥18.5, <24	52	0.99 (0.57, 1.74) 0.9779
≥24, <28	85	1.37 (1.02, 1.84) 0.0354
≥28	50	1.17 (0.78, 1.77) 0.4429
LDL-C, mmol/L		
<2.6	77	1.30 (0.93, 1.83) 0.1265
≥2.6	120	1.30 (0.99, 1.71) 0.0625
HDL-C, mmol/L		
<1.0	75	1.47 (1.02, 2.10) 0.0363
≥1.0	122	1.23 (0.94, 1.60) 0.1288
UA, μmol/L		
<420	125	1.14 (0.87, 1.49) 0.3505
≥420	72	1.68 (1.19, 2.38) 0.0030
RC quintiles		
Q1	6	0.43 (0.11, 1.77) 0.2453
Q2	12	0.64 (0.26, 1.58) 0.3337
Q3	35	1.31 (0.80, 2.14) 0.2871
Q4	51	1.69 (1.12, 2.56) 0.0132
Q5	93	1.48 (1.08, 2.02) 0.0147
Fatty liver		
No	97	1.18 (0.80, 1.73) 0.4101
Yes	100	1.36 (1.04, 1.77) 0.0231

Model 2: Adjusted for age, sex, BMI, ALT, AST, eGFR, TC, TG, HDL-C, LDL-C, UA, smoking habits, and drinking habits.

## Discussion

This is the first cohort study to compare the associations of RC and lipid parameters with HTN. In this large cohort study, we showed the interaction of RC with diabetes and risk factor management on subsequent risk of HTN incidence. We found that only elevated RC and TG ≥1.7 mmol/L were significantly associated with the subsequent risk of HTN after adjusting for various confounding factors. Specifically, diabetic people in the fifth quintile of RC had an approximately three-fold higher risk of HTN incidence than those in TG ≥ 1.7 mmol/L group. Compared with participants without diabetes, those with diabetes were more susceptible to having the excess risk of high RC on HTN incidence. In addition, the additional risk of HTN related to diabetes was obviously increased among participants with elevated RC. Further stratified analyses indicated that the deleterious effect on poor control of risk factors among people with diabetes would substantially increase the risk of HTN incidence. The findings of our study have significant implications that RC is an easy, simple, and significant predictor of HTN in the diabetic population, and targeting RC and blood glucose management are beneficial for incident HTN. Thus, early identification of HTN risk and management of HTN-related risk factors are of great significance.

It is well known that dyslipidemia has been regarded as the cornerstone of atherosclerosis and remains a significant risk factor for CVDs. Although LDL-C has been recommended as a crucial risk factor and therapeutic goal for CVDs in primary prevention, according to ACC/AHA and ESC/EAS guidelines (20), there is still an alarming number of adverse cardiovascular events regardless of desirable LDL-C–lowering therapies (28, 29), and meanwhile, emerging evidence proposed that RC may play a key role in the residual risk of CVDs (30). A growing number of studies suggested that increased RC levels are closely associated with the high risk of atherosclerosis and adverse events of CVDs (31). However, research focusing on the relationship between lipid metabolism and HTN is still lacking, particularly about RC. In a cohort study based on the Middle Eastern population, including 2,831 non-hypertensive women, TG and TG/HDL were found to be significantly predictive of incident HTN, and similar results were obtained from studies on adolescents (11, 12). More recently, a study of 5,173 participants reported that the increased RC level was significantly associated with higher central SBP, and this association was independent of other lipid levels. Our results showed the most significant association of elevated RC levels with incident HTN, which agrees with previous studies. Notably, despite LDL-C being the primary therapy target, it is not effectively predictive of HTN in our study. Numerous clinical studies supported the notion that a considerable residual risk of cardiovascular events still exists even when LDL-C is reduced at optimal values (28). RC as the cholesterol content of partially lipolytic TGRLs may be an important contributor to this residual risk to some extent (32, 33).

Clinically, the concentrations of TG serve as a surrogate indicator of TGRLs and RC. More recently, genetic and clinical intervention studies reported that RC is valuable in predicting the risk of CVDs, and RC but not LDL-C has been proven to be associated with cardiovascular events (34). This randomized controlled trial also indicated the stronger predictive ability of RC for cardiovascular events than TG. Our results reached similar conclusions on the association of RC and TG with HTN. People with TG ≥ 2.3 mmol/L have a 1.17-fold risk, and those with increased RC in the fifth quintile have a 1.69-fold risk of incident HTN, indicating the more significant predictive value of RC for incident HTN. In addition, in the hypertensive population, increased RC was significantly associated with albuminuria, which is proven a risk factor for CVDs (35), and this finding can further support our conclusions. High HDL-C has been considered to be protective of atherosclerosis-related to incident HTN, whereas low HDL-C contributed to the increased risk of CVDs (36). Interestingly, Crosby et al. reported that no causal relationship between low HLD-C and atherosclerosis was observed, and it is just an indicator of increased TGRLs concentration (37). Theoretically, HDL particles can penetrate the media after entering the intima and then leave the arterial wall by the lymphatic vessels and vascular wall of the outer membrane (38, 39). Conversely, remnants of TGRLs may be too large to penetrate the medium, leading to being trapped in the intima. The activity of LPL at the surface of remnants causes the increased liberation of free fatty acids and foam cell formation, contributing to vascular damage and inflammation (40). It might account for the stronger predictive value of RC for HTN; in addition, environmental and genetic factors can influence the results. Of note, although RC has not been uniformly defined, the calculated RC has been confirmed to possess equal credibility to directly RC in predicting CVDs risk in clinical practice (41). Given the convenience and highly cost-effective, RC calculated by the Friedewald formula has been widely promoted to be used in real-world clinical practice.

Notably, our results also supported a bidirectional interaction between RC and diabetes on incident HTN. RC and diabetes contribute independently to the increased risk of HTN, and among the diabetic population, RC is a modifiable risk factor for HTN. Given the considerable prevalence of dyslipidemia, diabetes, and HTN, the specific effect of high RC levels in the diabetic population deserves more attention. Studies exploring the interaction between RC and diabetes on incident HTN are scarce, and no research reported that RC is more valuable in predicting the subsequent risk of HTN among participants with diabetes than among those without diabetes. In this current study, we proposed novel findings that RC is a more significant predictor of incident HTN than TG or LDL-C in people with diabetes, and the interaction between RC and diabetes would amplify the deleterious effect of each other on subsequent risk of HTN. Our findings underline the importance of co-management of RC and blood glucose for the effective reduction of HTN incidence to prevent CVD events in the future. In addition, our findings supported the effects of ideal control of diabetes with risk factors on incident HTN is quite favorable. However, the HTN risk remained in people with high RC even with optimal blood glucose management. Theoretically, both dyslipidemia and diabetes contribute to vascular dysfunction by inducing endothelial injury and the formation of foam cells, and these shared pathogenic pathways may explain the interaction between RC and diabetes. These results suggested that using RC clinically to identify individuals at high risk of HTN might be beneficial. It should be emphasized that the management of blood pressure is strengthened for individuals with increased RC, especially in people with diabetes.

In summary, our study firstly reported that RC would be valuable in predicting the development of HTN in a Chinese population with diabetes, considering RC is used to accurately reflect the concentrations of TGRLs. The association of RC with CVDs has been fully demonstrated, and it has an unignored influence on atherosclerotic plaque formation, generating the environment to develop HTN to some extent. Our study revealed the important value of identifying individuals at high risk of developing HTN, who often are neglected due to the targeted LDL-C or HDL-C levels. Taken together, it is of great importance to early monitor RC in clinical practice, especially in people with diabetes. More importantly, RC might be both a significant predictor and a new observation or potential target in the management of HTN in the diabetic population.

The present study had some limitations. First, RC levels are calculated rather than directly measured. Although the direct measurement of RC has been developed, it is still difficult to be operational for clinical practice. In addition, lipoprotein subclasses have been reported to provide more significant information on CVD risk than the determination of traditional lipid parameters alone (42), but lipoprotein subclasses were not measured in this study. Thus, further studies are expected to clarify the association between lipoprotein subclasses and CVDs. Second, our study only recruited the Chinese population, it remains uncertain whether our results could be generalized to other ethnic groups. Finally, although we adjusted for known confounding factors in cox regression analysis, we cannot deny a possible residual because we did not investigate other medications that might affect this association, although this study excluded subjects taking anti-hypertensive drugs.

## Conclusions

To conclude, our study was the first cohort study and showed that an elevated RC at baseline is independently predictive of the development of HTN in a Chinese population with diabetes. More importantly, RC and diabetes significantly interacted with each other in imposing the increased risk of incident HTN. The favorable effects of lipid, glucose, and cardiovascular risk factors co-management should be emphasized among people at high risk of HTN in clinical practice and we point out that RC could be a new potential target and efficient biomarker for predicting HTN. We pronounce that targeting increased RC in people with diabetes, who are at high risk of HTN, may contribute to the drug discovery in the field of CVDs.

## Data availability statement

The datasets used to support this study are not freely available due to participants’ privacy protection. Requests to access the datasets should be directed to wangguang@bjcyh.com.

## Ethics statement

This study was approved by the Ethics Committee of the Beijing Chao-Yang Hospital affiliated with Capital Medical University. Written informed consent was obtained before the study. The patients/participants provided their written informed consent to participate in this study.

## Author contributions

All authors have read and approved the final manuscript. JW and QS have contributed equally to this work and share the first authorship. SL and GW have contributed equally to this work and share the corresponding authorship. JW contributed to the conception and design of the study. JW and QS analyzed and interpreted the data. YA and JL were involved in the collection and interpretation of the data. JW drafted the article. JW, QS, YA, JL, SL, and GW revised and reviewed the article. SL and GW supervised the study. All authors contributed to the article and approved the submitted version.

## Acknowledgments

We would like to thank the participants in this study.

## Conflict of interest

The authors declare that the research was conducted in the absence of any commercial or financial relationships that could be construed as a potential conflict of interest.

## Publisher’s note

All claims expressed in this article are solely those of the authors and do not necessarily represent those of their affiliated organizations, or those of the publisher, the editors and the reviewers. Any product that may be evaluated in this article, or claim that may be made by its manufacturer, is not guaranteed or endorsed by the publisher.

## Abbreviations

ALT, alanine aminotransferase; AST, aspartate aminotransferase; ASCVD, atherosclerosis cardiovascular diseases; BMI, body mass index; CI, confidence intervals; CVDs, cardiovascular diseases; Cr, creatinine; DBP, diastolic blood pressure; eGFR, estimated glomerular filtration rate; FPG, fasting plasma glucose; HDL-C, high-density lipoprotein cholesterol; HTN, hypertension; HR, hazard ratios; LDL-C, Low-density lipoprotein cholesterol; Lp(a), lipoprotein(a); RC, remnant cholesterol; SBP, systolic blood pressure; SD, standard deviations; TC, total cholesterol; T2DM, type 2 diabetes mellitus; TG, triglyceride; TGRLs, TG-rich lipoproteins; UA, uric acid.

## References

[1] Benjamin EJ Virani SS Callaway CW Chamberlain AM Chang AR Cheng S. Heart disease and stroke statistics-2018 update: A report from the American heart association. Circulation (2018) 137(12):e67–e492. doi: 10.1161/cir.0000000000000558 29386200

[2] Brunström M Carlberg B. Association of blood pressure lowering with mortality and cardiovascular disease across blood pressure levels: A systematic review and meta-analysis. JAMA Intern Med (2018) 178(1):28–36. doi: 10.1001/jamainternmed.2017.6015 29131895 PMC5833509

[3] O'Meara JG Kardia SL Armon JJ Brown CA Boerwinkle E Turner ST. Ethnic and sex differences in the prevalence, treatment, and control of dyslipidemia among hypertensive adults in the GENOA study. Arch Intern Med (2004) 164(12):1313–8. doi: 10.1001/archinte.164.12.1313 15226165

[4] Kario K Saito I Kushiro T Teramukai S Ishikawa Y Mori Y. Home blood pressure and cardiovascular outcomes in patients during antihypertensive therapy: primary results of HONEST, a large-scale prospective, real-world observational study. Hypertension (2014) 64(5):989–96. doi: 10.1161/hypertensionaha.114.04262 25156169

[5] Ference BA Kastelein JJP Ray KK Ginsberg HN Chapman MJ Packard CJ. Association of triglyceride-lowering LPL variants and LDL-C-Lowering LDLR variants with risk of coronary heart disease. Jama (2019) 321(4):364–73. doi: 10.1001/jama.2018.20045 PMC6439767 30694319

[6] Sarwar N Sandhu MS Ricketts SL Butterworth AS Di Angelantonio E Boekholdt SM. Triglyceride-mediated pathways and coronary disease: collaborative analysis of 101 studies. Lancet (2010) 375(9726):1634–9. doi: 10.1016/s0140-6736(10)60545-4 PMC2867029 20452521

[7] Lewington S Whitlock G Clarke R Sherliker P Emberson J Halsey J. Blood cholesterol and vascular mortality by age, sex, and blood pressure: a meta-analysis of individual data from 61 prospective studies with 55,000 vascular deaths. Lancet (2007) 370(9602):1829–39. doi: 10.1016/s0140-6736(07)61778-4 18061058

[8] Erqou S Kaptoge S Perry PL Di Angelantonio E Thompson A White IR. Lipoprotein(a) concentration and the risk of coronary heart disease, stroke, and nonvascular mortality. Jama (2009) 302(4):412–23. doi: 10.1001/jama.2009.1063 PMC3272390 19622820

[9] Ference BA Ginsberg HN Graham I Ray KK Packard CJ Bruckert E. Low-density lipoproteins cause atherosclerotic cardiovascular disease. 1. evidence from genetic, epidemiologic, and clinical studies. a consensus statement from the European atherosclerosis society consensus panel. Eur Heart J (2017) 38(32):2459–72. doi: 10.1093/eurheartj/ehx144 PMC5837225 28444290

[10] Silverman MG Ference BA Im K Wiviott SD Giugliano RP Grundy SM. Association between lowering LDL-c and cardiovascular risk reduction among different therapeutic interventions: A systematic review and meta-analysis. Jama (2016) 316(12):1289–97. doi: 10.1001/jama.2016.13985 27673306

[11] Tohidi M Hatami M Hadaegh F Azizi F. Triglycerides and triglycerides to high-density lipoprotein cholesterol ratio are strong predictors of incident hypertension in middle Eastern women. J Hum Hypertens (2012) 26(9):525–32. doi: 10.1038/jhh.2011.70 21776016

[12] Urbina EM Khoury PR McCoy CE Dolan LM Daniels SR Kimball TR. Triglyceride to HDL-c ratio and increased arterial stiffness in children, adolescents, and young adults. Pediatrics (2013) 131(4):e1082–90. doi: 10.1542/peds.2012-1726 PMC3608484 23460684

[13] De Backer G Jankowski P Kotseva K Mirrakhimov E Reiner Ž Rydén L. Management of dyslipidaemia in patients with coronary heart disease: Results from the ESC-EORP EUROASPIRE V survey in 27 countries. Atherosclerosis (2019) 285:135–46. doi: 10.1016/j.atherosclerosis.2019.03.014 31054483

[14] Mora S Wenger NK Demicco DA Breazna A Boekholdt SM Arsenault BJ. Determinants of residual risk in secondary prevention patients treated with high- versus low-dose statin therapy: the treating to new targets (TNT) study. Circulation (2012) 125(16):1979–87. doi: 10.1161/circulationaha.111.088591 PMC3338158 22461416

[15] Chapman MJ Ginsberg HN Amarenco P Andreotti F Borén J Catapano AL. Triglyceride-rich lipoproteins and high-density lipoprotein cholesterol in patients at high risk of cardiovascular disease: evidence and guidance for management. Eur Heart J (2011) 32(11):1345–61. doi: 10.1093/eurheartj/ehr112 PMC3105250 21531743

[16] Fruchart JC Sacks F Hermans MP Assmann G Brown WV Ceska R. The residual risk reduction initiative: a call to action to reduce residual vascular risk in patients with dyslipidemia. Am J Cardiol (2008) 102(10 Suppl):1k–34k. doi: 10.1016/s0002-9149(08)01833-x 19068318

[17] Jepsen AM Langsted A Varbo A Bang LE Kamstrup PR Nordestgaard BG. Increased remnant cholesterol explains part of residual risk of all-cause mortality in 5414 patients with ischemic heart disease. Clin Chem (2016) 62(4):593–604. doi: 10.1373/clinchem.2015.253757 26888894

[18] Varbo A Nordestgaard BG Tybjaerg-Hansen A Schnohr P Jensen GB Benn M. Nonfasting triglycerides, cholesterol, and ischemic stroke in the general population. Ann Neurol (2011) 69(4):628–34. doi: 10.1002/ana.22384 21337605

[19] Jørgensen AB Frikke-Schmidt R West AS Grande P Nordestgaard BG Tybjærg-Hansen A. Genetically elevated non-fasting triglycerides and calculated remnant cholesterol as causal risk factors for myocardial infarction. Eur Heart J (2013) 34(24):1826–33. doi: 10.1093/eurheartj/ehs431 23248205

[20] Mach F Baigent C Catapano AL Koskinas KC Casula M Badimon L. 2019 ESC/EAS guidelines for the management of dyslipidaemias: lipid modification to reduce cardiovascular risk. Eur Heart J (2020) 41(1):111–88. doi: 10.1093/eurheartj/ehz455 31504418

[21] Varbo A Nordestgaard BG. Remnant lipoproteins. Curr Opin Lipidol (2017) 28(4):300–7. doi: 10.1097/mol.0000000000000429 28548974

[22] Sun H Chang X Bian N An Y Liu J Leng S. Adipose tissue insulin resistance is positively associated with serum uric acid levels and hyperuricemia in northern Chinese adults. Front Endocrinol (Lausanne) (2022) 13:835154. doi: 10.3389/fendo.2022.835154 35757425 PMC9226335

[23] Levey AS Stevens LA Schmid CH Zhang YL Castro AF 3rd Feldman HI. A new equation to estimate glomerular filtration rate. Ann Intern Med (2009) 150(9):604–12. doi: 10.7326/0003-4819-150-9-200905050-00006 PMC2763564 19414839

[24] Bermudez-Lopez M Forne C Amigo N Bozic M Arroyo D Bretones T. An in-depth analysis shows a hidden atherogenic lipoprotein profile in non-diabetic chronic kidney disease patients. Expert Opin Ther Targets (2019) 23(7):619–30. doi: 10.1080/14728222.2019.1620206 31100024

[25] 2016 Chinese guidelines for the management of dyslipidemia in adults. J Geriatr Cardiol (2018) 15(1):1–29. doi: 10.11909/j.issn.1671-5411.2018.01.011 29434622 PMC5803534

[26] Jin C Chen S Vaidya A Wu Y Wu Z Hu FB. Longitudinal change in fasting blood glucose and myocardial infarction risk in a population without diabetes. Diabetes Care (2017) 40(11):1565–72. doi: 10.2337/dc17-0610 PMC5652588 28887409

[27] Zhou BF. Predictive values of body mass index and waist circumference for risk factors of certain related diseases in Chinese adults–study on optimal cut-off points of body mass index and waist circumference in Chinese adults. BioMed Environ Sci (2002) 15(1):83–96. doi: 10.1046/j.1440-6047.11.s8.9.x 12046553

[28] Twickler TB Dallinga-Thie GM Cohn JS Chapman MJ. Elevated remnant-like particle cholesterol concentration: a characteristic feature of the atherogenic lipoprotein phenotype. Circulation (2004) 109(16):1918–25. doi: 10.1161/01.Cir.0000125278.58527.F3 15117861

[29] Mihaylova B Emberson J Blackwell L Keech A Simes J Barnes EH. The effects of lowering LDL cholesterol with statin therapy in people at low risk of vascular disease: meta-analysis of individual data from 27 randomised trials. Lancet (2012) 380(9841):581–90. doi: 10.1016/s0140-6736(12)60367-5 PMC3437972 22607822

[30] Sandesara PB Virani SS Fazio S Shapiro MD. The forgotten lipids: Triglycerides, remnant cholesterol, and atherosclerotic cardiovascular disease risk. Endocr Rev (2019) 40(2):537–57. doi: 10.1210/er.2018-00184 PMC6416708 30312399

[31] Hong LF Yan XN Lu ZH Fan Y Ye F Wu Q. Predictive value of non-fasting remnant cholesterol for short-term outcome of diabetics with new-onset stable coronary artery disease. Lipids Health Dis (2017) 16(1):7. doi: 10.1186/s12944-017-0410-0 28086966 PMC5237249

[32] Varbo A Benn M Nordestgaard BG. Remnant cholesterol as a cause of ischemic heart disease: evidence, definition, measurement, atherogenicity, high risk patients, and present and future treatment. Pharmacol Ther (2014) 141(3):358–67. doi: 10.1016/j.pharmthera.2013.11.008 24287311

[33] Goliasch G Wiesbauer F Blessberger H Demyanets S Wojta J Huber K. Premature myocardial infarction is strongly associated with increased levels of remnant cholesterol. J Clin Lipidol (2015) 9(6):801–806.e1. doi: 10.1016/j.jacl.2015.08.009 26687701

[34] Castañer O Pintó X Subirana I Amor AJ Ros E Hernáez Á. Remnant cholesterol, not LDL cholesterol, is associated with incident cardiovascular disease. J Am Coll Cardiol (2020) 76(23):2712–24. doi: 10.1016/j.jacc.2020.10.008 33272365

[35] Li B Wang A Wang Y Li L Li B Yang Z. A study on the correlation between remnant cholesterol and urinary albumin to creatinine ratio in Chinese community adults: A report from the REACTION study. J Diabetes (2020) 12(12):870–80. doi: 10.1111/1753-0407.13076 32500969

[36] Nordestgaard BG Langsted A Freiberg JJ. Nonfasting hyperlipidemia and cardiovascular disease. Curr Drug Targets (2009) 10(4):328–35. doi: 10.2174/138945009787846434 19355857

[37] Crosby J Peloso GM Auer PL Crosslin DR Stitziel NO Lange LA. Loss-of-function mutations in APOC3, triglycerides, and coronary disease. N Engl J Med (2014) 371(1):22–31. doi: 10.1056/NEJMoa1307095 24941081 PMC4180269

[38] Rutledge JC Mullick AE Gardner G Goldberg IJ. Direct visualization of lipid deposition and reverse lipid transport in a perfused artery : roles of VLDL and HDL. Circ Res (2000) 86(7):768–73. doi: 10.1161/01.res.86.7.768 10764410

[39] Nordestgaard BG Hjelms E Stender S Kjeldsen K. Different efflux pathways for high and low density lipoproteins from porcine aortic intima. Arteriosclerosis (1990) 10(3):477–85. doi: 10.1161/01.atv.10.3.477 2344303

[40] Wahl P Ducasa GM Fornoni A. Systemic and renal lipids in kidney disease development and progression. Am J Physiol Renal Physiol (2016) 310(6):F433–45. doi: 10.1152/ajprenal.00375.2015 PMC4971889 26697982

[41] Cao YX Zhang HW Jin JL Liu HH Zhang Y Gao Y. The longitudinal association of remnant cholesterol with cardiovascular outcomes in patients with diabetes and pre-diabetes. Cardiovasc Diabetol (2020) 19(1):104. doi: 10.1186/s12933-020-01076-7 32631321 PMC7339517

[42] Klisic A Kavaric N Vujcic S Mihajlovic M Zeljkovic A Ivanisevic J. Inverse association between serum endocan levels and small LDL and HDL particles in patients with type 2 diabetes mellitus. Eur Rev Med Pharmacol Sci (2020) 24(15):8127–35. doi: 10.26355/eurrev_202008_22499 32767341

